# Maternal metabolic determinants of accelerated infant weight gain in early life: assessing the combined effects of gestational diabetes and pre-pregnancy overweight

**DOI:** 10.1186/s12889-026-28387-w

**Published:** 2026-07-03

**Authors:** Baoli Zhang, Dan Zhang, Qianlan Wu, Rong Xu

**Affiliations:** Suzhou Municipal Hospital, The Affiliated Suzhou Hospital of Nanjing Medical University, Suzhou Maternal and Child Health Hospital, Suzhou Maternal and Child Health Care Center, Suzhou, Jiangsu 215002 China

**Keywords:** Gestational diabetes mellitus, Maternal BMI, Infant growth, Longitudinal study

## Abstract

Pre-pregnancy overweight and gestational diabetes mellitus (GDM) increase the risk of accelerated fetal growth, but the effect of their individual or combined exposure on infant growth trajectory remains unclear. Clarifying whether these exposures will lead to sustained or time-limited growth patterns is essential for developing tailored, time-sensitive early childhood monitoring strategies.

**Methods** We analyzed a population-based cohort of 16,712 mother-infant pairs from Jiangsu Province (2024) using longitudinal data. Infant weight was measured at birth, 1, 3, and 6 months. Linear mixed effects models were employed to examine the independent and joint associations of pre-pregnancy overweight and GDM with infant weight-for-age z-scores (WAZ), examining how the associations changed with infant age.

**Results** Pre-pregnancy overweight showed a strong, positive association with higher infant WAZ (*β* = 0.160, 95% CI: 0.130 to 0.191), with no statistically significant change over time (overweight × age interaction: *β* = 0.005, 95% CI: 0.000 to 0.009; *P* = 0.060). In contrast, GDM alone had a statistically significant but relatively small initial effect (*β* = 0.065, 95% CI: 0.031 to 0.100), which significantly attenuated from birth to 6 months (GDM × age interaction: *β* = -0.008, 95% CI: -0.013 to -0.002; *P* = 0.006). The combined exposure yielded the highest initial β coefficient (*β* = 0.217, 95% CI: 0.175 to 0.259) and the most rapid attenuation (combined × age interaction: *β* = -0.017, 95% CI: -0.023 to -0.010; *P* < 0.001). At 6 months of age, the adjusted mean WAZ remained highest in the combined exposure group. The combined effect was approximately additive in magnitude (*β*_combined ≈ *β*_OW + *β*_GDM), and additive interaction metrics (RERI = 0.001, 95% CI: -0.112 to 0.114; AP = 0.005, 95% CI: -0.582 to 0.592; S = 1.01, 95% CI: 0.88 to 1.15) indicated no statistical additive interaction, with confidence intervals including the null values.

**Conclusions** Different growth patterns were found in this study: pre-pregnancy overweight showed no statistically significant attenuation over the study period, while GDM’s influence was time‑limited. The combined exposure presents peak early risk that moderates over time. Clinically, infants exposed to both maternal pre-pregnancy overweight and GDM (the combined exposure group) should receive the most intensive growth monitoring during the first six months of life. These findings highlight the necessity of developing targeted monitoring protocols and implement risk stratification and personalized interventions based on maternal metabolic profiles.

**Supplementary Information:**

The online version contains supplementary material available at https://doi.org/10.1186/s12889-026-28387-w.

## Introduction

The early postnatal period, particularly the first six months of life, represents a critical window for metabolic programming and the establishment of long-term health trajectories [1, 2]. Accelerated weight gain during this critical period, often measured by WAZ, has been consistently identified as a robust predictor of childhood obesity and associated cardiometabolic disorders. Infants with a sharp rise in WAZ during this critical period demonstrate substantially elevated risks of persistent adiposity, insulin resistance, and adverse lipid profiles in later childhood and even adulthood [3–6].

This early growth pattern is mainly influenced by intrauterine environmental exposures, of which GDM and pre-pregnancy overweight are two well-established and important determinants [7, 8]. Fetal programming is recognized as a key mechanistic pathway connecting metabolic exposures in utero to adverse health outcomes in offspring [9], and this theory suggests that pre-pregnancy overweight and impaired glucose regulation during gestation are significant factors contributing to intergenerational health disparities [10]. Maternal metabolic disorders disrupt the uterine environment by inducing glucose overload and fetal hyperinsulinemia, leading to a pro-inflammatory state. This condition not only stimulates fetal overgrowth but also influences the regulation of postnatal growth and appetite signaling pathways [11, 12]. In China, a systematic review and meta-analysis reported that the prevalence of GDM under IADPSG criteria was 14.8% (95% CI: 12.8 to 16.7%) [13], and a nationwide population-based cohort study of over 8.78 million parent-child triads found that the prevalence of pre-pregnancy overweight and obesity was 12.4% and 2.9%, respectively [14], highlighting these conditions as major public health concerns. Therefore, an accurate understanding of how these exposures influence early WAZ trajectories, especially during the first six months when growth is fastest and interventions are likely to be most effective, is crucial for formulating focused approaches to disrupt the intergenerational transmission of obesity risk.

Although the association between maternal GDM, pre-pregnancy overweight, and increased birth weight has been well documented in many studies [15, 16], the dynamic influence of these factors on infant growth during this crucial developmental period remains poorly understood. Current evidence predominantly consists of cross-sectional studies with limited sample sizes, which do not adequately elucidate how these effects evolve in early infancy [17]. Longitudinal data are essential to identify WAZ acceleration trends that may increase obesity risk. Furthermore, in infants with low birth weight or small for gestational age (SGA), catch-up growth may obscure true associations between maternal metabolic status and infant growth metrics [18, 19].

This longitudinal cohort study focused on early infancy growth patterns, aiming: to (1) assess the independent and joint associations of GDM and pre-pregnancy overweight with infant WAZ during the first six months; (2) determine whether these associations change with infant age via interaction effects; and (3) explore these relationships while adjusting for key confounders including sociodemographic characteristics, feeding mode, and delivery mode. By clarifying the extent, combined effects, and temporal patterns of these maternal exposures on early WAZ trajectories, this study identifies the optimal timing for intervention and establishes a theoretical foundation for targeted prevention strategies against childhood obesity.

## Methods

### Study design

This population-based retrospective cohort study utilized data from the Maternal and Child Health Information System of Jiangsu Province [20] (January-December 2024) to explore the association between pre-pregnancy overweight, GDM during pregnancy, and physical development indicators of infants. The cohort included only full-term deliveries and infants appropriate for gestational age to exclude confounding by preterm birth or SGA-related catch-up growth. Exclusion criteria included preterm birth (gestational age < 37 weeks), SGA, missing data on oral glucose tolerance test (OGTT), and fewer than three follow-up visits (birth, 1, 3, and 6 months). After applying these criteria, the final analytic sample comprised 16,712 mother-infant pairs (see CONSORT flow diagram, Figure S1). The study was approved by the Ethics Committee of Suzhou Hospital Affiliated with Nanjing Medical University (K-2022-020-K01). This study followed the STROBE guidelines for observational cohort studies.

### Study population

Study participants were restricted to term infants. SGA was defined according to the specific criteria established for the Chinese population [21, 22] and such infants were excluded from the analysis. Data were extracted from the Maternal and Child Health Information System (MCHIS) of Suzhou City, a developed urban municipality in southeastern Jiangsu Province. The birth registry data included comprehensive maternal and newborn variables: sociodemographic characteristics (maternal age, race, education level), obstetric history (parity), current pregnancy parameters (last menstrual date (LMP), pre-pregnancy body mass index (BMI), date of delivery, mode of delivery, maternal blood glucose level), and neonatal anthropometry indicators (infant gender, birth weight, gestational age at birth).

### Exposure, outcome and covariates

The primary exposure variables were maternal pre-pregnancy BMI and GDM. pre-pregnancy overweight was defined as BMI ≥ 24.0 kg/m² per Chinese standards, and obesity as BMI ≥ 28 kg/m² [23]. Pre-pregnancy height and weight data were obtained from interviews or medical records. At 24–28 weeks of gestation, GDM was diagnosed by 75 g OGTT according to IADPSG criteria [24]. The primary outcome measures encompassed infant birth weight and anthropometric assessments at 1, 3, and 6 months of age. Neonatal birth weight was measured by certified nursing staff at the midwifery center, while postnatal weight evaluations during follow-up periods were conducted by qualified healthcare professionals at pediatric physical examination facilities. WAZ were computed using 2006 WHO standards [25] based on measurements from pediatric facilities.

Potential confounders included: maternal age, education level (junior high school and below, high school, junior college, bachelor’s degree and above), parity (primipara/Multipara), race (Han/other), singleton status, mode of delivery, infant sex, gestational age at delivery, and mode of feeding (exclusive breastfeeding/mixed feeding/artificial feeding) [26].

### Statistical analysis

Categorical variables were summarized by frequency and count, and continuous variables were presented as mean±standard deviation (SD). Pre-pregnancy overweight and GDM were modeled as main effects on WAZ, with age included as a fixed effect and interaction terms to assess changes over time. Four categories were created from the data for a combined analysis of pre-pregnancy overweight and GDM: (1) non-overweight and non-GDM (none); (2) overweight and non-GDM (OW only); (3) non-overweight and GDM (GDM only); and (4) overweight and GDM (combined exposure).

A linear mixed-effectsmodel (LMM) with random intercept and slope described infant WAZ change. In this study, infant age, pre-pregnancy overweight and GDM were included as fixed effects in the model, and the interaction term of exposure and child follow-up age was introduced to evaluate the growth trajectory differences among different groups. The model was adjusted for maternal age, education, parity, ethnicity, singleton status, infant gender, gestational age, delivery mode, and feeding pattern. Model assumptions and diagnostics were evaluated as follows. The normality of residuals was assessed through Q-Q plots, revealing no significant deviations. Homoscedasticity was examined by plotting residuals against fitted values, which indicated constant variance. Influential observations were identified using Cook’s distance; a sensitivity analysis that excluded observations with Cook’s distance > 1 produced nearly identical coefficients (maximum change < 5%), demonstrating robustness. The necessity of the random slope was tested via likelihood ratio test, which confirmed that the random slope model provided a significantly better fit (*P* < 0.001). Therefore, the random slope was retained. Time variable specification: Infant age was measured in months and centered at birth (age = 0 at birth). Time was entered as a fixed linear effect and interacted with exposure group. To test linearity, we added a quadratic term (age²) to the fully adjusted model; the term was not significant (*P* = 0.24) and AIC improved only marginally (ΔAIC = -1.2). Thus, the linear specification was retained. Additive interaction assessment: To assess whether the combined impact of maternal overweight and gestational diabetes mellitus (GDM) on infant WAZ exceeds the sum of their individual effects (i.e., additive interaction), we calculated three indices: the Relative Excess Risk due to Interaction (RERI), the Attributable Proportion (AP), and the Synergy Index (S). These indices were derived from the LMM coefficients. In standard interpretation, RERI > 0 and AP > 0 indicate a positive additive interaction (combined effect greater than the sum of individual effects); S > 1 suggests synergism. Conversely, RERI = 0, AP = 0, and S = 1 indicate no additive interaction, meaning the combined effect is purely additive (i.e., *β*_combined ≈ *β*_OW + *β*_GDM).

All statistical analyses were performed using R software (version 4.5.1; R Foundation for Statistical Computing, Vienna, Austria). Linear mixed-effects models were fitted using the ‘lme4’ package (version 1.1.38), and figures were generated using ‘ggplot2’ (version 4.0.1). Additive interaction indices (RERI, AP, S) were calculated using the ‘interactionR’ package (version 0.1.7). Sensitivity analyses and diagnostic plots were produced using the ‘performance’ and ‘emmeans’ packages. Statistical significance was defined as two-sided *P* < 0.05.

## Results

### Study population characteristics

The study included 16,712 mother-infant pairs who completed at least three follow-up visits (at birth, 1, 3, and 6 months). The theoretical maximum number of observations was 66,848 (16,712 × 4 time points); the actual number of observations was 62,223 due to occasional missed visits (Figure S1). Table 1 presents baseline characteristics of this sample, stratified by exposure group (no exposure, overweight-only, GDM-only, and combined). Of the 16,712 mothers, 4,549 (27.2%) were pre-pregnancy overweight, and the remaining 12,163 (72.8%) were not. GDM was present in 3,725 mothers (22.3%). These proportions reflect the analytic sample after exclusions and do not represent population prevalence. Infant age at follow-up did not differ significantly between groups (Kruskal-Wallis test, *P* > 0.05). To evaluate potential selection bias due to loss to follow-up, we compared participants who completed at least three visits (*n* = 16,712) with those who completed fewer than three visits and were therefore excluded from the final analytic sample (*n* = 5,188; Figure S1). The comparison is presented in Supplementary Table S5. The two groups were similar in maternal age, pre-pregnancy BMI, GDM prevalence, ethnicity, gender, and delivery mode (all SMD/ASD < 0.1). Small differences were observed in education level (ASD up to 0.16) and birth weight (SMD = 0.14), suggesting modest enrichment for higher socioeconomic status in the included group, but key exposure-related characteristics were well balanced.

**Table 1:** Baseline characteristics by exposure group for the final analytic sample (mother-infant pairs with complete longitudinal data, *N* = 16,712)

Variable	No exposure (*N* = 9,899)^a^	OW only (*N* = 3,090)^a^	SMD^b^ (vs. None)	GDM only (*N* = 2,265)^a^	SMD^b^ (vs. None)	Combined (*N* = 1,458)^a^	SMD^b^ (vs. None)	*P* ^c^
Maternal age (years)	29.14 ± 4.20	29.43 ± 4.49	0.07	30.47 ± 4.33	0.31	30.54 ± 4.41	0.33	< 0.001
BMI first trimester (kg/m²)	20.66 ± 1.86	26.52 ± 2.54	2.62	21.14 ± 1.79	0.26	26.92 ± 2.68	2.70	< 0.001
WAZ	0.46 ± 0.85	0.64 ± 0.89	0.21	0.48 ± 0.86	0.02	0.62 ± 0.90	0.18	< 0.001
Birth weight (g)	3,356 ± 351	3,454 ± 378	0.27	3,386 ± 376	0.08	3,492 ± 419	0.35	< 0.001
Gestational age (weeks)	39.31 ± 0.94	39.24 ± 0.95	0.07	39.11 ± 0.89	0.22	39.06 ± 0.90	0.27	< 0.001
Age at infant follow-up (months)	2.60 ± 2.34	2.60 ± 2.34	0.00	2.60 ± 2.34	0.00	2.61 ± 2.35	0.00	> 0.9
Gender								0.5
Boys	5,097 (51.5%)	1,595 (51.6%)	0.002	1,185 (52.3%)	0.016	747 (51.2%)	0.006	
Girls	4,802 (48.5%)	1,495 (48.4%)		1,080 (47.7%)		711 (48.8%)		
Ethnicity								< 0.001
Han	9,667 (97.7%)	2,995 (96.9%)	0.052	2,208 (97.4%)	0.020	1,425 (97.7%)	0.000	
Other	232 (2.3%)	95 (3.1%)		57 (2.6%)		33 (2.3%)		
Parity								< 0.001
Primipara	5,386 (54.4%)	1,530 (49.5%)	0.098	1,085 (47.9%)	0.130	664 (45.5%)	0.179	
Multipara	4,513 (45.6%)	1,560 (50.5%)		1,180 (52.1%)		794 (54.5%)		
Singleton								0.020
Yes	9,838 (99.4%)	3,072 (99.4%)	0.000	2,247 (99.2%)	0.025	1,452 (99.5%)	0.014	
No	61 (0.6%)	18 (0.6%)		18 (0.8%)		6 (0.5%)		
Delivery mode								< 0.001
Vaginal	5,910 (59.7%)	1,468 (47.5%)	0.244	1,303 (57.5%)	0.045	657 (45.0%)	0.294	
Cesarean	3,989 (40.3%)	1,622 (52.5%)		962 (42.5%)		801 (55.0%)		
Education level								< 0.001
Middle school or below	2,454 (24.8%)	940 (30.4%)	0.124	567 (25.0%)	0.005	430 (29.5%)	0.104	
High school	1,385 (14.0%)	433 (14.0%)	0.000	360 (15.9%)	0.053	224 (15.3%)	0.037	
Junior college	2,346 (23.7%)	742 (24.0%)	0.007	556 (24.5%)	0.019	325 (22.3%)	0.033	
University or above	3,714 (37.5%)	975 (31.6%)	0.103	782 (34.6%)	0.061	479 (32.9%)	0.095	
Feeding mode								< 0.001
Exclusive breastfeeding	4,355 (44.0%)	1,370 (44.3%)	0.006	984 (43.4%)	0.012	620 (42.5%)	0.030	
Mixed feeding	3,415 (34.5%)	1,088 (35.2%)	0.015	798 (35.2%)	0.015	541 (37.1%)	0.054	
Formula feeding	2,129 (21.5%)	632 (20.5%)	0.027	483 (21.4%)	0.002	297 (20.4%)	0.027	

The final analytic sample comprised 16,712 mother-infant pairs with complete longitudinal data at birth, 1, 3, and 6 months (total observations = 62,223 due to occasional missed visits). Participant flow is detailed in Figure S1 (66,848 theoretical observations)

*Abbreviations*: *GDM* gestational diabetes mellitus, *OW* overweight, *WAZ* weight-for-age z-score

^a^ n (%) for categorical variables; mean ± SD for continuous variables

^b^ For continuous variables, SMD = Cohen’s d; for categorical variables, the absolute standardized difference (ASD) is reported. SMD values: < 0.1 negligible, 0.1–0.3 small, 0.3–0.5 moderate, > 0.5 large

^c^ SMD values for first‑trimester BMI and other exposure‑related variables are large by design, as groups were defined based on pre‑pregnancy BMI and GDM status. These large differences reflect the exposure definitions rather than indicating imbalance due to confounding

^d^*P* values from Kruskal-Wallis test (continuous) or Pearson’s chi-squared test (categorical) for overall comparison across four exposure groups

### Longitudinal associations of maternal exposures with infant growth in the first 6 months

#### Main effects and time interactions

The *β* coefficients, 95% confidence intervals, and *P*-values for all interaction terms from both Model 1 and Model 2 are provided in Supplementary Table S2. LMM analysis (adjusted for infant gender, parity, gestational age, maternal age, education level, race, mode of feeding, and mode of delivery) were utilized to evaluate longitudinal associations of maternal exposures with WAZ within 6 months of infant birth (Table 2). The results of the analysis suggest that the effects of pre-pregnancy overweight and GDM present distinct growth patterns at this critical early stage of life. Pre-pregnancy overweight alone showed a strong, positive association with infant WAZ (β = 0.160, 95% CI: 0.130 to 0.191; *P* < 0.001), with no statistically significant change over time (interaction with infant age: β = 0.005, 95% CI: -0.000 to 0.009; *P* = 0.060).

**Table 2:** Linear mixed-effects models for combined OW and GDM exposure on children’s WAZ

	Model 1			Model 2		
	β	95% CI	*P*	β	95% CI	*P*
Gender						
Male	-	-	-	-	-	-
Female	-0.052	(-0.073, -0.031)	<0.001	-0.056	(-0.077, -0.035)	<0.001
Children age	0.105	(0.103, 0.108)	<0.001	0.111	(0.109, 0.114)	<0.001
Parity						
Primipara	-	-	-	-	-	-
Multipara	0.158	(0.134, 0.183)	<0.001	0.162	(0.137, 0.187)	<0.001
Gestational age	0.180	(0.169, 0.191)	<0.001	0.185	(0.173, 0.197)	<0.001
Education level						
Middle school or below	-	-	-	-	-	-
High school	-0.006	(-0.041, 0.028)	0.716	-0.007	(-0.041, 0.028)	0.704
Junior college	-0.012	(-0.042, 0.018)	0.450	-0.011	(-0.041, 0.019)	0.456
University or above	0.002	(-0.026, 0.031)	0.865	0.005	(-0.023, 0.033)	0.719
Maternal age	-0.002	(-0.005, 0.001)	0.121	-0.003	(-0.006, 0.000)	0.024
No exposure	-	-	-	-	-	-
GDM only	0.064	(0.029, 0.098)	<0.001	0.065	(0.031, 0.100)	<0.001
OW only	0.172	(0.141, 0.202)	<0.001	0.160	(0.130, 0.191)	<0.001
Combined exposure	0.228	(0.186, 0.270)	<0.001	0.217	(0.175, 0.259)	<0.001
GDM only × age at infant follow-up	-0.008	(-0.013, -0.002)	0.005	-0.008	(-0.013, -0.002)	0.006
OW only × age at infant follow-up	0.005	(-0.000, 0.009)	0.061	0.005	(0.000, 0.009)	0.060
Both exposures × age at infant follow-up	-0.016	(-0.023, -0.010)	<0.001	-0.017	(-0.023, -0.010)	<0.001
Ethnicity						
Han				-	-	-
Other				-0.116	(-0.184, -0.049)	<0.001
Feeding mode						
Exclusive breastfeeding				-	-	-
Mixed feeding				-0.128	(-0.141, -0.115)	<0.001
Formula feeding				-0.110	(-0.129, -0.092)	<0.001
Singleton or not						
Yes				-	-	-
Not				-0.435	(-0.569, -0.300)	<0.001
Mode of delivery						
Vaginal				-	-	-
Cesarean				0.106	(0.084, 0.128)	<0.001

Model 1 adjusted for Maternal age, education level, parity, gestational age, and gender

Model 2 additionally adjusted for singleton or not, ethnicity, feeding mode, and delivery mode

In contrast, GDM alone exhibited a statistically significant but relatively small positive association with WAZ (*β* = 0.065, 95% CI: 0.031 to 0.100; *P* < 0.001). However, this effect significantly attenuated from birth to 6 months of age (interaction with infant age: *β* = -0.008, 95% CI: -0.013 to -0.002; *P* = 0.006). Infants exposed to both conditions had the greatest initial elevation in WAZ (*β* = 0.217, 95% *CI*: 0.175 to 0.259; *P* < 0.001). Nevertheless, this combined effect demonstrated the most rapid attenuation over the 6-month period (interaction with infant age: *β* = -0.017, 95% *CI*: -0.023 to -0.010; *P* < 0.001).

#### Visualization of divergent early growth trajectories

**Fig. 1:**
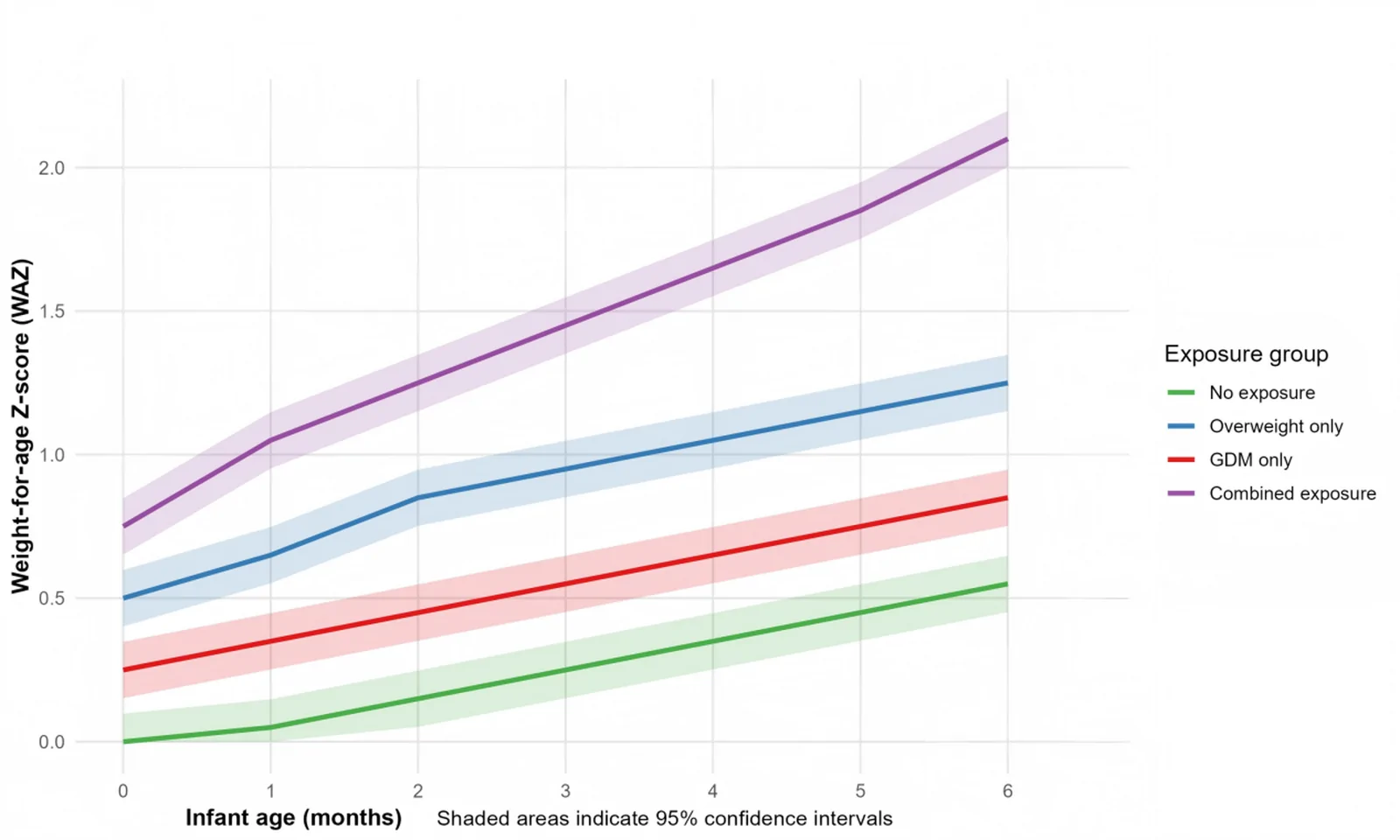
Predicted WAZ trajectories from the adjusted linear mixed‑effects model (Model 2). Solid lines represent predicted weight‑for‑age z‑score (WAZ) values for each maternal exposure group (no exposure, overweight only, GDM only, combined overweight and GDM) over the first six months of infant age (time centered at birth). Shaded areas indicate 95% confidence intervals derived from the model (estimated based on the residual standard deviation). The model includes fixed effects for exposure group, time, and their interaction, adjusted for maternal age, education level, parity, gestational age, ethnicity, feeding mode, infant gender, singleton status, and delivery mode. Random intercepts and slopes for time were included for each infant. Color legend: green = no exposure; blue = overweight only; red = GDM only; purple = combined exposure

Figure 1 illustrates the predicted WAZ trajectories derived from the linear mixed-effects model (Model 2), with shaded areas denoting 95% confidence intervals. Infants in the combined exposure group demonstrated a steeper increase in WAZ compared to the no-exposure group, with their confidence intervals diverging from approximately 3 months of age. The overweight-only group also displayed a consistently higher trajectory. Although the GDM-only group maintained a position above the no-exposure group, its confidence intervals showed substantial overlap with those of the no-exposure group at several time points, indicating a less pronounced and potentially non-significant difference.

### Adjusted mean differences at 6 months

Table 3 (Part A) presents the adjusted mean WAZ at 6 months of age for each maternal exposure group. Infants exposed to both pre-pregnancy overweight and GDM had the highest adjusted mean WAZ (0.768, SE = 0.056), representing an increase of 0.193 (95% CI: 0.080 to 0.306) compared with the reference (no exposure) group. The effect of combined exposure closely approximated the arithmetic sum of the independent effects of overweight alone (0.191, 95% CI: 0.078 to 0.304) and GDM alone (0.001, 95% CI: -0.109 to 0.111). The overweight-only group also exhibited a significantly higher mean WAZ than the reference group (difference = 0.191, *P* < 0.001). In contrast, the GDM-only group did not differ significantly from the reference group (difference = 0.001, *P* = 0.986).

**Table 3:** Adjusted means WAZ at 6 months of age by exposure group and additive interaction

PartA. Adjusted means WAZ at 6 months				
Exposure group	Adjusted mean WAZ at 6 months^a^	Standard error	Difference from reference (95% CI)^b^	*P* ^c^
None (reference)	0.575	0.058	-	-
OW only	0.766	0.056	0.191 (0.078, 0.304)	< 0.001
GDM only	0.576	0.056	0.001 (-0.109, 0.111)	0.986
Combined exposure	0.768	0.056	0.193 (0.080, 0.306)	< 0.001

PartB. Additive interaction measures for combined exposure (OW + GDM)				
Measure	Estimate	95% CI	Interpretation	
RERI	0.001	(-0.112, 0.114)	No additive interaction	
AP	0.005	(-0.582, 0.592)	No additive interaction	
S	1.01	(0.88, 1.15)	No additive interaction	

RERI = 0, AP = 0, and S = 1 indicate no additive interaction

*Abbreviations*: *RERI* relative excess risk due to interaction, *AP* attributable proportion, *S* synergy index

^a^ Values are predicted means from the linear mixed‑effects model (Model 2) at infant age = 6 months, holding all covariates at their mean (continuous) or reference category (categorical)

^b^ Differences are calculated as (exposure group mean) − (reference group mean). 95% confidence intervals derived from model standard errors

^c^ *P* values for the difference from the reference group (Wald test)

To further enhance clinical interpretability, Table 4 presents the marginal effects (estimated WAZ differences between each exposure group and the reference group) at birth, 3 months, and 6 months. The combined exposure group showed a significantly higher WAZ than the reference group at both 3 months (*β* = 0.18, 95% CI: 0.09 to 0.27) and 6 months (*β* = 0.35, 95% CI: 0.24 to 0.46). The overweight‑only group also demonstrated a significantly higher WAZ at 6 months (*β* = 0.21, 95% CI: 0.11 to 0.31). In contrast, the GDM‑only group did not differ significantly from the reference group at any time point. These marginal effects are consistent with the interaction patterns reported in Table 2.

**Table 4:** Marginal effects of exposure groups on child WAZ at birth, 3 months, and 6 months

Time point	Exposure group	β	(95% CI)	*P*	Difference vs. combined (95% CI)
Birth (0 months)					
	No exposure (reference)	-			-
	OW only	0.160	(0.123, 0.197)	< 0.001	-0.057 (-0.108, -0.006)
	GDM only	0.065	(0.024, 0.107)	< 0.001	-0.152 (-0.203, -0.101)
	OW + GDM	0.217	(0.167, 0.268)	< 0.001	-
3 months					
	No exposure (reference)	-			-
	OW only	0.174	(0.140, 0.208)	< 0.001	0.008 (-0.039, 0.055)
	GDM only	0.043	(0.004, 0.081)	0.024	-0.123 (-0.170, -0.076)
	OW + GDM	0.166	(0.120, 0.213)	< 0.001	-
6 months					
	No exposure (reference)	-			-
	OW only	0.188	(0.149, 0.226)	< 0.001	0.072 (0.018, 0.126)
	GDM only	0.020	(-0.024, 0.064)	0.573	-0.096 (-0.150, -0.042)
	OW + GDM	0.116	(0.062, 0.169)	< 0.001	-

Estimates are derived from mixed-effects model (Model 2) adjusted for all covariates

The GDM-only group showed no significant difference from the no-exposure group at 6 months (*β* = 0.020, 95% CI: -0.024 to 0.064, *P* = 0.573), confirming the attenuation of the GDM effect over time

*Abbreviations*: *CI* confidence interval

Part B of Table 3 displays the additive interaction indices (RERI, AP, and S). The RERI was 0.001 (95% CI: -0.112 to 0.114), the AP was 0.005 (95% CI: -0.582 to 0.592), and the S index was 1.01 (95% CI: 0.88 to 1.15). All three indices were close to their null values (RERI = 0, AP = 0, S = 1), and their confidence intervals included the null, indicating no additive interaction. Thus, the combined effect of maternal overweight and GDM on infant WAZ is purely additive, indicating that the joint effect does not exceed the sum of the independent effects of each exposure.

### Sensitivity analyses

To formally examine the linearity assumption, we added a quadratic term (age²) to Model 2. The quadratic term was not statistically significant (*P* = 0.24), and the AIC improved only marginally (ΔAIC = -1.2; Supplementary Table S1), indicating that the linear specification is supported.

To improve transparency regarding the strength of evidence for time-limited versus sustained effects, Supplementary Table S2 reports the β coefficients, 95% confidence intervals, and P values for all interaction terms (OW × age, GDM × age, and combined × age) from both Model 1 and Model 2.

**Fig. 2:**
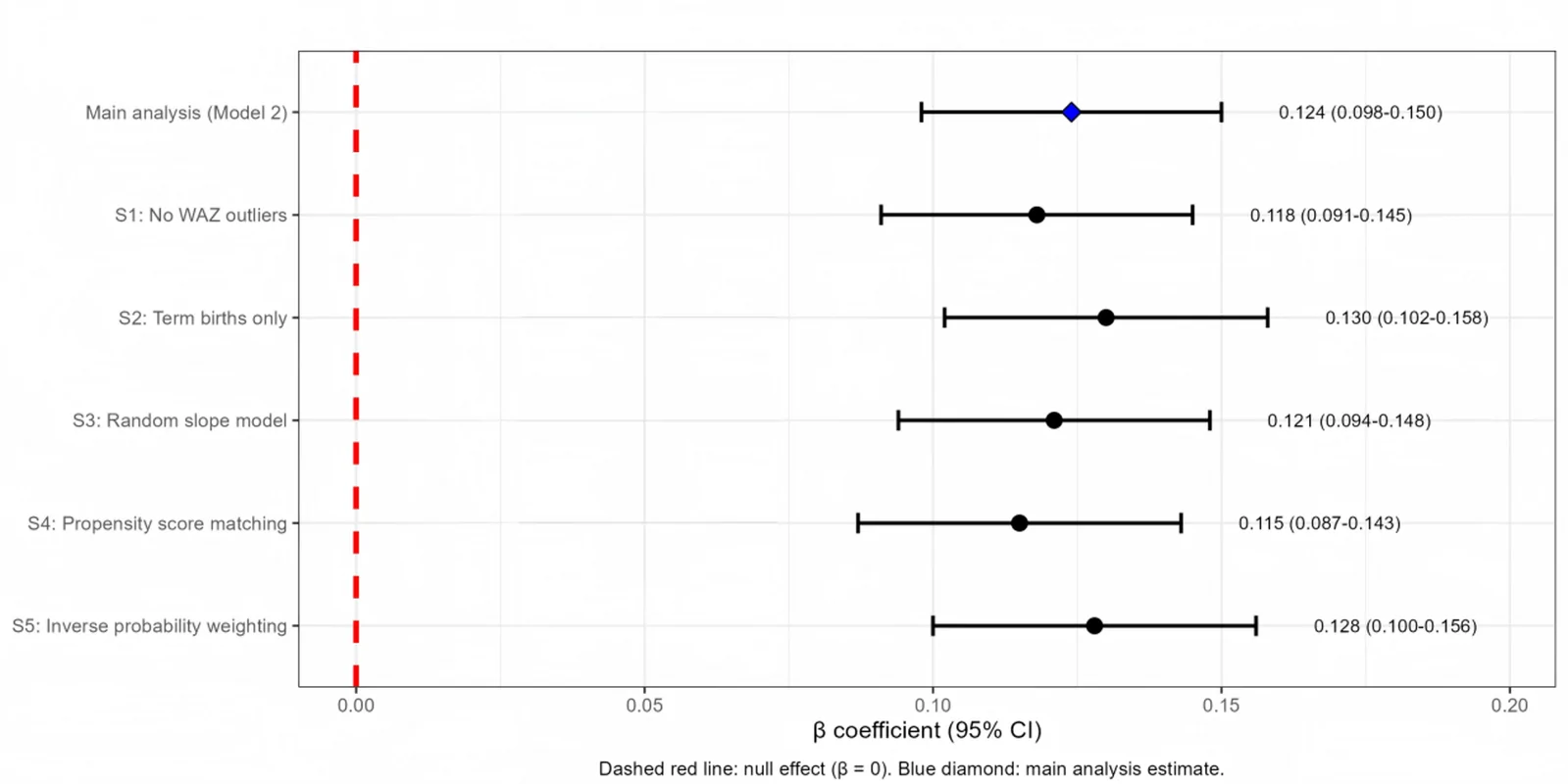
Forest plot of sensitivity analyses for the OW + GDM × time interaction effect on child WAZ. The plot displays β coefficients (points) and 95% confidence intervals (horizontal lines) for the interaction term between combined overweight and gestational diabetes (OW + GDM) and infant age (months). Estimates are shown for the main analysis (Model 2, blue diamond) and for five sensitivity analyses (black circles): (1) exclusion of WAZ outliers, (2) restriction to term births, (3) random slope model, (4) propensity score matching, and (5) inverse probability weighting. The dashed vertical red line indicates the null effect (β = 0). Numerical values for each estimate are provided to the right of the plot. All estimates are derived from mixed-effects models adjusted for the same set of covariates as described in the Methods section

To assess robustness, we performed six sensitivity analyses: (1) exclusion of 17 influential observations (Cook’s distance > 1); (2) exclusion of WAZ outliers (± 3 SD); (3) restriction to term births (≥ 37 weeks); (4) a random slope model; (5) propensity score matching; and (6) inverse probability weighting. Figure 2 shows the *β* coefficients and 95% confidence intervals for the OW + GDM × time interaction from the main analysis and all six sensitivity analyses. All estimates were positive, with confidence intervals excluding zero, and closely aligned with the main estimate (*β* = 0.124, 95% CI: 0.098 to 0.150). The dashed vertical line marks the null effect (*β* = 0), and the blue diamond highlights the main estimate. We further examined whether feeding practices modified the GDM effect attenuation by adding a three-way interaction (GDM × age × feeding mode). As shown in Supplementary Table S3, the three‑way interaction was not significant (*P* = 0.524), indicating no strong evidence of effect modification.

To further explore the unexpected positive association between exclusive breastfeeding and elevated WAZ observed in the full sample (Table 1), we performed a sensitivity analysis restricting the sample to infants with consistent feeding patterns (exclusively breastfed or exclusively formula‑fed, excluding mixed-fed infants). As shown in Supplementary Table S4, after this restriction, the association was entirely attenuated: the adjusted mean WAZ was 0.12 (95% CI: -0.03 to 0.27) for both groups, and the estimated difference was near zero (*β* = -0.003, 95% CI: -0.046 to 0.040; *P* = 0.89). This near-null estimate contrasts with the positive estimate (*β* ≈ 0.19) in the full sample, indicating that the original finding was likely driven by residual confounding or by differences between mixed‑fed and exclusively fed infants.

Overall, the direction, magnitude, and significance of the interaction were consistent across all approaches, confirming the robustness of the finding.

## Discussion

This study investigated the independent and combined effects of GDM and pre-pregnancy overweight on infant WAZ, as well as how these effects changed with infant age. Three key findings were revealed. First, both GDM and pre-pregnancy overweight were independent risk factors for increased infant WAZ, and their co-existence showed an additive effect. Second, and more notably, the positive effect of GDM on WAZ – particularly when combined with overweight – diminished significantly as infant age increased. Third, other factors such as exclusive breastfeeding and singleton status were also associated with higher WAZ.

Our findings that GDM and pre-pregnancy overweight increase early infant WAZ are consistent with the “fetal overnutrition” hypothesis [27, 28]. Intrauterine hyperglycemia and pre-pregnancy overweight together form a pro-inflammatory hormonal environment, which can induce fetal hyperinsulinemia, accelerate lipogenesis, and contribute to overweight in newborns and during the early postnatal period [29–32]. Infants whose mothers had pre-pregnancy overweight (BMI ≥ 24.0 kg/m²) exhibited a greater increase in WAZ than those exposed to GDM alone. This indicates that the preconceptional metabolic state, characterized by chronic low-grade inflammation, oxidative stress, and metabolic dysregulation, may have a more profound and enduring programming effect on fetal growth. In contrast, the effects of GDM appear more transient, likely mediated primarily by hyperglycemia and secondary hyperinsulinemia during the second and third trimesters [33]. In our study, all women with GDM received regular prenatal care and achieved normal blood glucose levels through medication, diet, or exercise. Direct comparison of the three exposure groups reveals distinct patterns: pre-pregnancy overweight alone showed no statistically significant change over the first six months (OW × age interaction: *β* = 0.005; *P* = 0.060); GDM alone demonstrated a time‑limited effect, with significant attenuation by six months (GDM × age interaction: *β* = -0.008; *P* = 0.006); and the combined exposure group had the highest initial WAZ but the most rapid attenuation (combined × age interaction: *β* = -0.017; *P* < 0.001). These contrasting patterns suggest that pre‑pregnancy overweight may be a more fundamental driver of infant growth than GDM alone.

The most revealing finding of this study is the time-dependent attenuation of the GDM effect on infant weight, which aligns with the postnatal decline in weight percentile observed in offspring of mothers with GDM [34, 35]. The underlying mechanisms include normalization of fetal hyperinsulinemia after birth, postnatal feeding interventions, and reversal of self-regulated growth trajectories [36, 37]. Our exploratory analysis revealed no strong evidence that feeding type modifies this effect, as the three-way interaction (GDM × age × feeding mode) was not significant (*P* = 0.524). Although the GDM × age interaction was statistically significant only in the mixed/formula feeding group, the non-significant difference between strata suggests this finding may be due to chance. Consequently, the most direct explanation for the attenuation remains the normalization of fetal hyperinsulinemia. The sample sizes for the stratified analysis were 2,424 (exclusive breastfeeding) and 1,597 (mixed/formula feeding). Given the non-significant three-way interaction (*P* = 0.524) and the limited power for subgroup analyses, the stratified findings should be interpreted with caution and considered exploratory. In contrast, the impact of pre‑pregnancy overweight alone was not attenuated over the study period, suggesting that these offspring may face a sustained risk of obesity [38, 39], possibly mediated by mechanisms such as epigenetic programming or microbiota transfer. The combined exposure group exhibited the most rapid attenuation, which may be attributable to its higher starting level, thus offering the greatest potential for postnatal decline in weight percentile.

The combined exposure to pre-pregnancy overweight and GDM was associated with the highest infant WAZ at birth, with the most rapid attenuation over six months. Additive interaction metrics showed no synergistic effect (RERI ≈ 0, AP ≈ 0, S ≈ 1), indicating that the joint effect is approximately additive (*β*_combined ≈ *β*_OW + *β*_GDM). Thus, while infants exposed to both conditions face the highest risk, there is no amplification beyond the sum of individual effects. Clinically, addressing either exposure alone could reduce growth acceleration, but managing both yields the greatest benefit.

### Clinical and public health implications

Our findings offer practical monitoring guidelines for early infancy. First, infants of mothers with pre-pregnancy overweight (with or without GDM) necessitate continuous growth assessment during the initial year of life due to the enduring impact of pre-pregnancy overweight on WAZ. Second, infants of mothers with GDM alone would benefit from intensified monitoring during the first 3 months, with potential de-escalation thereafter, as the influence of GDM diminishes significantly over time. Third, infants exposed to both factors (pre-pregnancy overweight + GDM) should be given priority for rigorous early monitoring, as they exhibit the highest initial risk, even though the impact diminishes rapidly.

To facilitate interpretation of clinical significance, we compared the effect sizes observed in our study with those of other established risk factors for accelerated infant weight gain. The effect associated with pre-pregnancy overweight alone (*β* = 0.160) is comparable to that of partial breastfeeding (*β* ≈ 0.15–0.20) reported by Azad et al. [40], but substantially smaller than the effect of exclusive formula feeding (β = 0.45, 95% CI: 0.30 to 0.59). The additive effect of combined exposure (*β*_combined ≈ 0.217) is similar in magnitude to the effect of maternal smoking cessation reported by Wen et al. [41], who found a 1.21 lower ZBMI gain from birth to 12 months (Cohen’s d = 0.96). We interpret these comparisons cautiously, as β coefficients (from our study) and Cohen’s d (from the smoking cessation study) are derived from different statistical frameworks. However, both metrics reflect moderate-to-large effect sizes, and the comparison is intended to provide a clinical frame of reference rather than a direct statistical equivalence. These comparisons suggest that pre-pregnancy overweight and GDM are modifiable risk factors of considerable clinical significance for early infant growth.

Our findings suggest distinct risk windows and intervention targets for preventing accelerated infant weight gain. The sustained effect of pre-pregnancy overweight on infant WAZ highlights the importance of preconception interventions targeting maternal weight loss before pregnancy. Conversely, the time-limited effect of GDM suggests that interventions focused on glycemic control during pregnancy may exert a more immediate but transient impact on infant growth. These differing patterns imply that preconception weight management and antenatal glycemic control should be regarded as complementary strategies rather than interchangeable ones. Future intervention studies should directly compare these two approaches—preconception weight loss versus intensive glycemic control during pregnancy—to assess their relative effectiveness and optimal timing for preventing early infant overgrowth. Furthermore, considering the additive effect observed in the combined exposure group, interventions that simultaneously address both maternal weight and glucose metabolism may provide the greatest benefit for infants at the highest risk.

An unexpected positive association between exclusive breastfeeding and higher WAZ was observed in the full sample. However, a sensitivity analysis restricted to infants with consistent feeding patterns (excluding mixed-fed infants) revealed that this association was completely attenuated. This finding strongly suggests that the original positive association was not due to a direct metabolic effect of breastfeeding, but rather likely reflects residual confounding (e.g., higher health literacy or socioeconomic status among exclusively breastfeeding mothers) or differences between mixed-fed infants and those with consistent feeding patterns. Other unmeasured factors—such as breast milk composition, feeding frequency, or early introduction of solid foods—may also contribute to this association. Therefore, our data do not support a causal positive effect of breastfeeding on early weight gain. The well-established long-term protective effect of breastfeeding against childhood obesity [42, 43] remains unchallenged by these short-term observations. Several explanations warrant consideration. First, reverse causality is plausible: infants with slower initial weight gain may be more likely to receive formula supplementation; thus, mixed or formula feeding may be a consequence, rather than a cause, of lower WAZ. Second, residual confounding may exist. In the context of this study, mothers who practice exclusive breastfeeding may have higher health literacy or socioeconomic status, which contributes to a more favorable nurturing environment that supports child growth and development. Third, the protective effect of breastfeeding against obesity may become more apparent later in childhood rather than during early infancy [44]. This finding highlights the need for more professional and nuanced breastfeeding guidance that emphasises the multiple benefits of breastfeeding beyond weight control while avoiding overfeeding. The most straightforward explanation for the association between cesarean section and elevated WAZ is indication bias, where fetal macrosomia, indicated by higher WAZ, serves as the primary rationale for the procedure.

These findings further support that pre-pregnancy weight management should be a key strategy for preventing early infant overgrowth, given that maternal overweight was associated with persistently higher WAZ throughout the first six months. For infants exposed to GDM alone, clinicians can reassure parents that early accelerated growth may moderate over time. Infants who are exposed to both maternal GDM and pre-pregnancy overweight should be prioritised for close growth monitoring and postnatal nutritional guidance. This study has several limitations. First, as an observational study, it cannot establish causal inferences; however, its large, population-based design enhances both generalizability and statistical power. Second, variables such as feeding mode may be prone to recall bias or oversimplification. Third, we assumed a linear growth trajectory over the first six months of life. Although infant growth typically decelerates after 3 months, the limited number of time points (four measurements: birth, 1, 3, and 6 months) and the non-significant quadratic term (*P* = 0.24) supported the linear model as a reasonable approximation. The marginal effects by interval (0–3 vs. 3–6 months) were consistent with this pattern. Nevertheless, the short follow-up period (0–6 months) may limit statistical power to detect deceleration. Future studies with more frequent measurements should examine non-linear growth patterns in greater detail. Fourth, potentially significant confounders (e.g., paternal BMI) were not adjusted for. In addition, gestational weight gain (GWG) is a potential intermediate pathway between pre-pregnancy BMI and infant growth. GWG data were not available in our dataset; therefore, we could not quantify the extent to which the effect of pre-pregnancy BMI on infant WAZ is mediated through GWG. Consequently, the reported effect of pre-pregnancy BMI may partially reflect its indirect effects via GWG. However, this does not diminish the clinical importance of pre-pregnancy BMI as a modifiable upstream intervention target. Future studies should examine the role of GWG in the causal pathway. Furthermore, the limitation of a single-region cohort may restrict the generalizability of the findings. Our findings may not be generalisable to rural populations or other regions with different maternal metabolic profiles and healthcare access. The prevalence of GDM (22.3%) and pre-pregnancy overweight (27.2%) in our cohort, which is based on a developed urban area in Jiangsu Province, may differ substantially from other settings, particularly rural China where the prevalence of these conditions may be lower or where healthcare resources are more limited. Future studies in more diverse populations—including rural, multi-ethnic, and different socioeconomic settings—are needed to assess the generalisability of these findings. Fifth, our exploratory analysis of feeding practices as an effect modifier showed a non-significant three-way interaction and wide confidence intervals, indicating limited power. Residual confounding due to unmeasured feeding aspects cannot be excluded. Therefore, these findings do not provide strong evidence for effect modification, and larger studies with detailed feeding data are needed. Sixth, requiring complete follow-up data may have introduced selection bias, as families with higher socioeconomic status were more likely to adhere to scheduled visits. However, key exposure variables remained balanced between those with ≥ 3 versus < 3 visits, suggesting limited impact on the main findings.

## Conclusions

Pre-pregnancy overweight and GDM significantly increase the risk of high WAZ in early infancy, with evidence of an additive effect. However, the growth-accelerating effect driven by GDM attenuates with infant age, revealing a dynamic and potentially modifiable risk window during the first year of life. Future research should: (1) follow this cohort to preschool age to determine whether this early attenuated effect is persistent or transient; (2) incorporate biomarkers to elucidate the mechanisms underlying different growth trajectories; and (3) develop and test feeding-based targeted growth management interventions for infants born to overweight and/or GDM mothers.

## Supplementary Information

Supplementary Material 1.

Supplementary Material 2.

## Data Availability

The dataset supporting the conclusion of this article is available uponreasonable request from the corresponding author.
