# Supplementary material for: Maternal metabolic determinants of accelerated infant weight gain in early life: assessing the combined effects of gestational diabetes and pre-pregnancy overweight
Source: BMC Public Health. 2026 Jul 3;26:2661. doi: 10.1186/s12889-026-28387-w (PMC13602569; doi:10.1186/s12889-026-28387-w)
Supplement: Supplementary file 1 [file 12889_2026_28387_MOESM1_ESM.docx]

**Figure S1.** CONSORT‑style flow diagram of participant selection.
**Abbreviations:** OGTT, oral glucose tolerance test; GA, gestational age; SGA, small for gestational age.
