# Supplementary material for: Maternal metabolic determinants of accelerated infant weight gain in early life: assessing the combined effects of gestational diabetes and pre-pregnancy overweight
Source: BMC Public Health. 2026 Jul 3;26:2661. doi: 10.1186/s12889-026-28387-w (PMC13602569; doi:10.1186/s12889-026-28387-w)
Supplement: Supplementary file 2 [file 12889_2026_28387_MOESM2_ESM.docx]

**Supplementary Table S1.** Sensitivity analysis of combined exposure effects on infant WAZ across different model specifications

| **Analysis** | **Effect** | ***β* (95% *CI*)** | ***P*** |
| --- | --- | --- | --- |
| Main analysis | Combined exposure (main) | 0.217 (0.176 , 0.258) | <0.001 |
| Main analysis | Combined exposure × age | -0.017 (-0.023, -0.011) | <0.001 |
| Exclude extreme WAZ (±3 SD) | Combined exposure (main) | 0.211 (0.170, 0.252) | <0.001 |
| Exclude extreme WAZ (±3 SD) | Combined exposure × age | -0.020 (-0.026, -0.014) | <0.001 |
| Complete cases | Combined exposure (Main) | 0.217 (0.176, 0.258) | <0.001 |
| Complete cases | Combined exposure × age | -0.017 (-0.023, -0.011) | <0.001 |
| Random slope for age at infant follow-up | Combined exposure (main) | 0.227 (0.190, 0.264) | <0.001 |
| Random slope for age at infant follow-up | Combined exposure × age | -0.017 (-0.027, -0.007) | <0.001 |
| Reference: OW only | Combined exposure (main) | 0.057 (0.010, 0.104) | 0.018 |
| Reference: OW only | Combined exposure × age | -0.021 (-0.029, -0.013) | <0.001 |
| Linear model (main) | Age (linear) | 0.112 (0.109, 0.115) | <0.001 |
|  | Age × Combined exposure | -0.017 (-0.023, -0.011) | <0.001 |
| Quadratic model | Age (linear) | 0.108 (0.104, 0.112) | <0.001 |
|  | Age² (quadratic) | -0.002 (-0.005, 0.001) | 0.24 |
|  | Age² × Combined exposure | -0.001 (-0.004, 0.002) | 0.51 |

Note: Infant age was centered at birth (0 months). Models were adjusted for all covariates as in Model 2 (maternal age, education level, parity, gestational age, ethnicity, feeding mode, infant gender, singleton status, delivery mode); Note: AIC = Akaike information criterion; lower values indicate better fit. ΔAIC = -1.2 favours the quadratic model only marginally; the quadratic term was not statistically significant (*P* = 0.24)..

**Supplementary Table S2.** Interaction terms from linear mixed-effects models

| **Model** | **Interaction term** | **β (95% CI)** | **P** |
| --- | --- | --- | --- |
| Model 1 | OW only × age | 0.005 (-0.000, 0.009) | 0.055 |
| Model 1 | GDM only × age | -0.008 (-0.013, -0.003) | 0.004 |
| Model 1 | Combined exposure × age | -0.016 (-0.023, -0.010) | <0.001 |
| Model 2 | OW only × age | 0.005 (-0.002, 0.011) | 0.17 |
| Model 2 | GDM only × age | -0.008 (-0.015, -0.000) | 0.038 |
| Model 2 | Combined exposure × age | -0.017 (-0.026, -0.008) | <0.001 |

Note: Infant age was centered at birth (0 months). Model 1 adjusted for maternal age, education level, parity, gestational age, and infant gender. Model 2 additionally adjusted for ethnicity, feeding mode, singleton status, and delivery mode. P‑values < 0.001 are reported as '< 0.001'.

**Supplementary Table S3.** Effect modification of feeding practices on GDM-related WAZ attenuation

| **Feeding mode** | **GDM × age interaction (β)** | **95% CI** | ***P*** |
| --- | --- | --- | --- |
| Exclusive breastfeeding | -0.0088 | (-0.0189, 0.0013) | 0.087 |
| Mixed / Formula feeding | -0.0128 | (-0.0250, -0.0006) | 0.040 |
| Three-way interaction (difference between feeding modes) | -0.0040 | (-0.0164, 0.0084) | 0.524 |

*Note: GDM × age interaction represents the difference in monthly WAZ change between GDM-exposed and unexposed infants within each feeding stratum. The three-way interaction tests whether this difference varies by feeding mode. Models adjusted for all covariates as in Model 2. Feeding mode was treated as time-varying.*

**Supplementary Table S4.** Association between exclusive breastfeeding and WAZ in infants with consistent feeding patterns (excluding mixed-fed infants)

| **Feeding group** | **N** | **Adjusted mean WAZ (95% CI)** | ***β* (95% CI)** | ***P*** |
| --- | --- | --- | --- | --- |
| Exclusively formula-fed (reference) | 1,597 | 0.12 (-0.03, 0.27) | — | — |
| Exclusively breastfed | 2,424 | 0.12 (-0.03, 0.27) | — | — |
| Difference (breastfed vs. Formula-fed) | — | — | -0.003 (-0.046, 0.040) | 0.890 |

*Note: Mixed-fed infants (those who changed feeding mode or received mixed feeding at any time point) were excluded. Adjusted means and 95% confidence intervals were derived from a linear mixed‑effects model (Model 2) adjusted for maternal age, education level, parity, gestational age, ethnicity, infant gender, singleton status, and delivery mode. Confidence intervals for adjusted means are asymptotic due to large sample size. The near-zero difference (*β* = -0.003) and wide confidence intervals indicate no meaningful association between exclusive breastfeeding and WAZ in this restricted sample.*

**Supplementary Table S5.** Comparison of baseline characteristics between participants with ≥3 follow-up visits (included) and those with <3 follow-up visits (excluded)

| **Variable** | **Included**  **(≥3 visits, N = 16,712)** | **Excluded**  **(<3 visits, N = 5,188)** | **SMD/ASD¹** |
| --- | --- | --- | --- |
| Maternal age (years) | 29.5 ± 4.3 | 29.6 ± 4.6 | -0.02 |
| Pre-pregnancy BMI (kg/m²) | 22.3 ± 3.4 | 22.5 ± 3.5 | -0.06 |
| GDM, n (%) | 4,148 (22.5%) | 1,223 (23.6%) | 0.03 |
| Education, n (%) |  |  |  |
| - Middle school or below | 4,512 (24.5%) | 1,589 (30.7%) | 0.14 |
| - High school | 2,486 (13.5%) | 821 (15.9%) | 0.07 |
| - Junior college | 4,150 (22.5%) | 1,117 (21.6%) | 0.03 |
| - University or above | 6,043 (32.8%) | 1,331 (25.7%) | 0.16 |
| Han ethnicity, n (%) | 17,950 (97.3%) | 4,984 (96.3%) | 0.07 |
| Birth weight (g) | 3,317 ± 435 | 3,253 ± 548 | 0.14 |
| Male sex, n (%) | 9,586 (52.0%) | 2,727 (52.7%) | 0.01 |
| Cesarean section, n (%) | 10,183 (55.2%) | 2,806 (54.2%) | 0.02 |

¹ SMD = standardized mean difference (Cohen's d) for continuous variables; ASD = absolute standardized difference for categorical variables. SMD/ASD < 0.1 indicates negligible imbalance; 0.1-0.3 indicates small imbalance; > 0.3 indicates moderate to large imbalance.
Note: The 'Excluded' group comprises participants who had fewer than three follow-up visits. Missing values were excluded from the calculation of percentages.
